# The functional relations among motor-based prediction, sensory goals and feedback in learning non-native speech sounds: Evidence from adult Mandarin Chinese speakers with an auditory feedback masking paradigm

**DOI:** 10.1038/s41598-018-30399-5

**Published:** 2018-08-09

**Authors:** Xiaoluan Liu, Xing Tian

**Affiliations:** 1 New York University Shanghai, Shanghai, China; 2 Shanghai Key Laboratory of Brain Functional Genomics (Ministry of Education), School of Psychology and Cognitive Science, East China Normal University, Shanghai, China; 3 NYU-ECNU Institute of Brain and Cognitive Science, New York University Shanghai, Shanghai, China

## Abstract

Previous studies in speech production and acquisition have mainly focused on how feedback vs. goals and feedback vs. prediction regulate learning and speech control. The present study investigated the less studied mechanism–prediction vs. goals in the context of adult Mandarin speakers’ acquisition of non-native sounds, using an auditory feedback masking paradigm. Participants were asked to learn two types of non-native vowels: /ø/ and /ɵ/—the former being less similar than the latter to Mandarin vowels, either in feedback available or feedback masked conditions. The results show that there was no significant improvement in learning the two targets when auditory feedback was masked. This suggests that motor-based prediction could not directly compare with sensory goals for adult second language acquisition. Furthermore, auditory feedback can help achieve learning only if the competition between prediction and goals is minimal, i.e., when target sounds are distinct from existing sounds in one’s native speech. The results suggest motor-based prediction and sensory goals may share a similar neural representational format, which could result in a competing relation in neural recourses in speech learning. The feedback can conditionally overcome such interference between prediction and goals. Hence, the present study further probed the functional relations among key components (prediction, goals and feedback) of sensorimotor integration in speech learning.

## Introduction

In speech acquisition and control, three factors collectively contribute to the development and maintenance of accurate speech production: auditory feedback, motor-based prediction and sensory goals^1–4^. Considerable amount of scholarly effort has been spent on the investigation of relations between feedback vs. prediction and feedback vs. goals for speech learning and control. The present study is aimed at addressing the less studied issue, i.e., prediction vs. goals, through the lens of Mandarin Chinese adult acquisition of different types of non-native speech sounds.

### Auditory feedback, motor-based prediction and sensory goals in speech production

Auditory feedback has attracted considerable scholarly interest due to its importance in speech production. Previous research using empirical and modelling approaches to motor-sensory integration in speech production^5–7^ suggests that auditory feedback is mainly responsible for ensuring successful vocal achievement of speech targets because feedback enables comparison between the speaker’s own speech and the target speech. More specifically, feedback adjusts the motor control system according to online speech production in such a way that it corrects and updates the internal motor prediction system until speech errors are eliminated and target speech is achieved^8^.

Examples abound as to the importance of auditory feedback in speech production and acquisition. Hearing impairments in early life can severely affect children’s normal speech production and development^9^. Adults suffering from hearing loss also tend to encounter difficulties in maintaining accurate speech production, especially in terms of speech rate, intensity and F0^10,11^. Intelligibility of production of vowels and consonants is also affected if proper auditory feedback is deprived in adulthood^12^. When the hearing environment becomes less optimal (e.g., noise), the need for auditory feedback is reflected in the Lombard effect^13^, i.e., speakers’ involuntary vocal response to ambient noise by increasing vocal loudness.

However, due to the delay in neural processing of sensory feedback (e.g., neural conduction and central processing), effective control of motor movement also requires the involvement of motor-based prediction, i.e., an internally maintained representation of motor control which predicts consequences of motor movement^14^. In speech production, motor-based prediction (prediction henceforth) refers to the internal estimate of the current state of vocal tract dynamics and subsequent auditory results^3,15,16^. Such prediction is based on prior knowledge of the causal relation between speech motor commands and sensory output^1–4,15–20^. Particularly, the causal relation reflects a key dimension of motor learning (e.g., speech acquisition), the primary computation of which requires the formalization of the motor plan based on sensory targets (i.e., sensory-to-motor transformation). The establishment of such computation and verification of the correctness of sensory-to-motor transformation need an online estimation of how good the results will be based on the planned motor movement. Therefore, prediction about sensory consequences of the motor system is needed.

Prediction is hypothesized to be realized through a system termed internal forward model^1,7,21^ to estimate the possible outcome of articulatory movement before auditory feedback is received. Such mechanism thus enables fast online motor correction of non-standard articulatory output. Auditory feedback, meanwhile, serves to update and maintain the internal forward model^2^. Thus, prediction allows one to deal with complex speech situations where various extents of articulatory and perceptual demands could challenge accurate speech production^2,22^. Evidence for the existence of prediction can be found in perturbation studies where either speech articulation^23,24^ or perceptual feedback^25–27^ was artificially perturbed, e.g., by either shifting pitch or formants from a standard baseline. It was found that perturbation generally elicited a compensatory speech motor response opposite in direction to the manipulated shift in feedback. The observations imply that an internal prediction of the speech target is used to guide the online compensation for the artificially introduced feedback perturbation.

Both auditory feedback and prediction help monitor and verify the achievement of sensory goals, which are a major factor in speech production^3,28,29^. As Fairbanks^30^ postulated, the goal of speech production was represented by sensory outcomes. Specifically, sensory goals define the final target of speech production, and they could be stored in auditory and phonological system. Thus, the function of sensory goals in speech production is to activate speech targets stored in memory during speech acquisition^2^. The most obvious example of the existence of sensory goals is that children’s speech production patterns are determined by the acoustic input they obtain^2^. Adult speech production is also influenced by ambient speech because they tend to automatically reproduce phonetic patterns (e.g., pitch, vowel features) introduced in their surrounding acoustic environment^31^. Moreover, speakers with greater sensory acuity tend to produce larger phonological contrasts (e.g., vowels), which implies the recruitment of sensory goals in speech production^29^. In addition, several studies have shown the activation of auditory-related brain areas (e.g., posterior superior temporal sulcus/gyrus) in accessing phonological representations in speech production^32,33^. Sensory goals thus have been modeled as a major stage in speech production^1,3,5,34,35^.

Overall, auditory feedback, prediction and sensory goals are important factors in speech production. The relations between the factors in speech production have been captured in prominent theoretical models such as the Directions Into Velocities of Articulators (DIVA) model^1,28^, Task Dynamic (TD) model^36^, State Feedback Control (SFC) model^4^, and similar models such as those proposed in Tian & Poeppel^15,16^ and Hickok^3^. In those models, an efference copy is sent out to an internal model of the vocal tract by motor commands to predict the current state of the vocal tract. The sensory outcome of the motor commands is estimated by an additional auditory efference copy. Errors occur when there is a deviation between the predicted and actual sensory feedback, which will then be fed back to the internal model to guide motor movement until the goal is correctly achieved.

### Relations among prediction, goals, and feedback in adult acquisition of different types of non-native speech sounds

The mechanisms of feedback, prediction and goals in speech production discussed above are particularly evident in human vocal learning^1,2,5^: goals are compared with feedback in establishing the causal relation between motor commands and sensory output. Child L1 learning and adult L2 learning are two important domains of vocal learning. Compared with infants and children, adults tend to encounter more difficulties in learning the phonetic/phonological aspect of a foreign language^37,38^. From a sensorimotor perspective, such difficulties could be attributed to the already well-established sensory-to-motor and motor-to-sensory transformations (i.e. the mechanism for motor based prediction) in adults as a result of long-term practice of native language. Young children, on the other hand, usually do not achieve mature, adult-like motor control of speech production until 11 or 12 years of age^39^. Consequently, in early stages of native speech acquisition, auditory feedback is heavily relied upon to monitor speech production errors. Adults, on the contrary, rely more on motor-based prediction mechanisms in speech production due to accurate estimation of the causal relation between motor commands and sensory consequences^40,41^.

When it comes to learning a foreign language, the mature motor-sensory loops established in adults’ native language could become a barrier in effectively forming new motor-sensory transformation for the foreign language, hence the ‘critical period hypothesis’^42^ and ‘fundamental difference hypothesis’^43^ on the neurocognitive differences between child L1 and adult L2 learners. That helps explain why a high similarity between L1 and L2 sounds (i.e., an overlap in phonological space between L1 and L2) could hinder adult L2 learners’ ability to effectively establish the representation of L2 sounds^37,38,44^. The reason could lie in the difficulty in adjusting the already mature motor-to-sensory establishment for L1 to the new L2 sounds that are close in perception and production to L1 sounds. Supporting evidence mainly comes from speech perception studies. A typical example is that Japanese speakers tend to learn English /ɹ/ better than /l/ due to the fact that /ɹ/ is not as close as /l/ to their corresponding Japanese phonemes^45^. For speakers of a tonal language (e.g., Mandarin), acquisition of novel L2 tones may not be easy either. Studies have found that there does not necessarily exist a tone-language advantage in learning foreign tones^46–48^. For example, speakers of Hmong (a tonal language) were worse than English speakers in Mandarin tone identification^48^ (Wang, 2006). It was also found that Cantonese speakers, whose native language has six tones, still encountered difficulty in differentiating tonal contrasts not found in Cantonese^49^. These findings suggest that the native tonal system could exert a negative or even interfering influence on acquisition of non-native tones^48^.

### The present study: investigation of prediction vs. goals

In sum, the above review on motor control in speech learning suggests that a considerable amount of scholarly effort has been spent on the relations between feedback vs. goals and feedback vs. prediction. In terms of feedback vs. goals, the review in section 1.1 suggests that sensory goals are used to compare with auditory feedback in establishing both sensory-to-motor and motor-to-sensory transformations. The evidence can be found in empirical studies and computational modelling of children’s acquisition of native speech sounds, where comparison between auditory feedback and sensory goals serves to update motor commands for the achievement of speech targets^2,5^. With regard to feedback vs. prediction, studies on self-speech induced suppression (SIS)^18,50–52^ and feedback perturbation^26,27^ suggest that sensory feedback should match motor prediction in speech production and perception, otherwise an enhanced auditory response (e.g., as indexed by M100 which is an auditory response around 100 ms measured in magnetoencephalography) could be triggered to monitor and correct the deviation between the feedback and prediction.

Therefore, what remains less investigated is prediction vs. goals (i.e., the relation between prediction and goals) especially in the case of adults’ acquisition of non-native speech sounds with different degrees of similarity to their native speech. Therefore, the present study attempts to address the following question: can prediction compare directly with goals in speech acquisition? We investigate this question in the context of adults’ acquisition of L2, because as reviewed in section 1.2, the motor-based prediction is available via the established motor-to-sensory transformation in adults as a result of long-term practice of their native language. Thus, there could be a competing relation between L1 and L2 in terms of motor demand on speech production. Moreover, studies on adult L2 perception suggest that the extent of similarity between L1 and L2 sound targets correlates with the degree of difficulty for adults to accurately establish representations of L2 targets (goals), as reviewed in section 1.2. Thus, we further investigate how similarity between prediction established in L1 (via motor-sensory transformation) and goals in L2 (speech targets) affects adults’ speech learning.

To investigate the above research questions, in this study online auditory feedback was manipulated in two ways: feedback masked with noise (the *masked* condition) and without noise (the *unmasked* condition) when participants learned to produce two different vowels: /ø/ (e.g., in German) and /ɵ/ (e.g., in Dutch). The first vowel is less similar than the latter vowel to the closest existing pronunciation of Mandarin Chinese vowels (more details in the following sections). In the *masked* condition, participants can only rely on prediction and goals for speech learning. Given that the participants have not established a precise mapping between neuromuscular command for the articulatory trajectory (i.e., motor prediction) and desired sensory output for the new L2 sounds (i.e., sensory goals), the lack of auditory feedback would render the formation of such mapping more difficult, which could consequently limit their ability to directly compare between prediction and goals to calculate speech errors for update of motor commands.

Therefore, we hypothesize that prediction and goals cannot be directly compared for adult L2 speech learning, regardless of the type of L2 sounds. Thus, we predict that participants can learn neither of the two vowels when feedback is unavailable, i.e., in the *masked* condition. Whereas in the *unmasked* condition, auditory feedback is available for learning and we predict that the extent of similarity between prediction in L1 (via motor-sensory transformation) and speech targets in L2 (goals) will affect learning performance. More specifically, based on the above review, learning /ø/ should be better than learning /ɵ/ in the *unmasked* condition. This is because as shown in previous literature, a high degree of similarity between L1 and L2 speech sounds makes adult L2 acquisition more difficult (as reviewed in section 1.2). From a sensory-motor point of view, this could suggest that the more similarities between motor prediction for L1 and sensory goals in L2, the more interference there could be between them in adult L2 learning. The interference would render goals less accurate during the course of learning. Since /ɵ/ is more similar than /ø/ to existing pronunciations in Mandarin Chinese, there would be more interference from participants’ native language (i.e., motor prediction established through L1) in maintaining the goal for /ɵ/ than for /ø/, which as a result would yield auditory feedback less effective in improving the learning performance of /ɵ/.

Two experiments were conducted to test the above predictions. Experiment 1 used a within-subject design while Experiment 2 used a between-subject design with two additional listening control tasks. A between-subject design was needed to test whether the results of the within-subject design can be replicated. Moreover, a between-subject design could filter out the practice effect which often results from a within-subject repeated measures design. Two additional listening tasks were needed to control for perception and memory accuracy which could be confounds. Specifically, we ruled out the possibility that inaccurate perception and memory of the target speech sounds could lead to the participants’ bad performance of speech production.

## Experiment 1

### Methods

The study was approved by NYU Shanghai Research Ethical Committee. All experiments were performed according to relevant guidelines and regulations. Informed consent was obtained from all participants for the experiments. Data sets are accessible from https://osf.io/cnvh7/.

#### Stimuli

**Figure 1:**
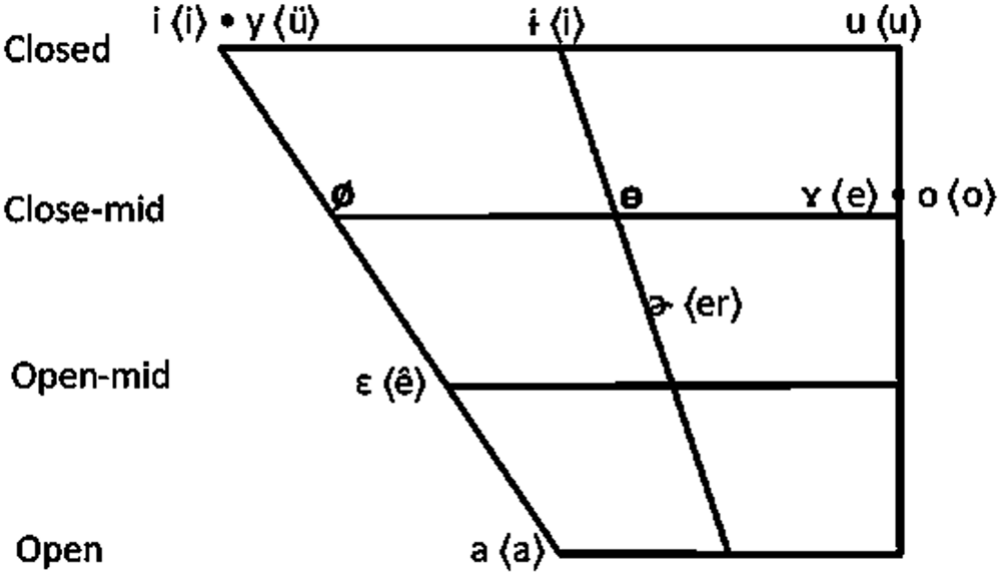
IPA and Pinyin chart for Mandarin vowels and the two target vowels /ø/ and /ɵ/.

Two vowels were selected as stimuli: /ø/ and /ɵ/. This is because they represent different degrees of similarity to Mandarin vowels, which is a key factor in this study as discussed in the Introduction section. Specifically, /ɵ/ sounds perceptually similar to existing Mandarin vowels /ɤ/ and /ɚ/ (Fig. 1), while /ø/ is harder to be perceptually related to an existing Mandarin vowel.

The stimuli were recorded by a male and female native speaker of the respective language (German for /ø/ and Dutch for /ɵ/) in a sound-attenuated booth. They were asked to produce the stimuli five times in their native language. Then they picked one token of their recording that they were most satisfied with, which were used as stimuli for this study. Each stimulus was repeated 10 times (i.e., 10 tokens of the same stimulus) in this study. The average duration of the two vowels were 500 ms.

To justify our selection of the two vowels as stimuli, a production and a perception validation tests were conducted. We invited twelve Mandarin speakers (age *M* = 28, SD = 3. 8, six females) to produce the seven Mandarin vowels (/a/, /o/, /ɤ/, /i/, /u/, /y/, /ɚ/) prompted on a computer screen, with each vowel produced three times. The participants were then asked to reproduce the two target vowels /ø/ and /ɵ/ delivered auditorily, three times for each vowel. Then they were asked to perceptually compare the two target vowels with their own production of the Mandarin vowels by rating the perceptual similarity between the target vowels and each one of the Mandarin vowels on a scale of 0 to 5, in which 0 means ‘not similar at all’, 5 means ‘very similar’, with the rest of the numbers meaning somewhere in-between the two extremes.

The results were consistent with our expectation. In terms of production, the mean Euclidean distance between /ø/ and existing Mandarin vowels produced by the participants was 2.82 (*SEM* = 0.08), which was larger than the Euclidean distance between /ɵ/ and existing Mandarin vowels (*M* = 2.58, *SEM* = 0.05), and the difference was significant (*F* (1, 11) = 16.72, *p* < 0.001, *η*^2^_p_ = 0.6). A linear mixed model was carried out with gender treated as a fixed factor, individual speaker as a random factor, and F1 and F2 raw Hertz was used to calculate the Euclidean distance between Mandarin vowels and the two target sounds /ø/ and /ɵ/. The results showed that gender significantly affected the results in the /ø/ condition (*F* (1, 10) = 35.7, *p* < 0.001), and the /ɵ/ condition (*F* (1, 10) = 13, *p* = 0.005). Specifically, females had higher Euclidean distance than males in both conditions (for /ø/, females: *M* = 597.11 Hz, *SEM* = 18.55 Hz; males: *M* = 432.75 Hz, *SEM* = 20.3 Hz; for /ɵ/, females: *M* = 521.13 Hz, *SEM* = 17.22 Hz; males: *M* = 435.74 Hz, *SEM* = 14.62 Hz).

With regard to the perception test, the results showed that on average, the similarity rating score was significantly higher for /ɵ/ (*M* = 1.74, *SEM* = 0.1) than for /ø/ (*M* = 0.93, *SEM* = 0.08) [F (1, 11) = 54.34, p < 0.001, *η*^2^_p_ = 0.83)], suggesting participants found /ɵ/ to be more similar to existing Mandarin vowels than /ø/. A closer look at the data revealed that participants particularly found /ɵ/ to be similar to Mandarin vowel /ɤ/ (average rating score: 4.56) and /ɚ/ (average rating score: 4.25). Therefore, the production and perception tests support our selection of /ø/ and /ɵ/ as representing different degrees of similarity to existing Mandarin vowels.

#### Participants

Seventeen adult native speakers of Mandarin Chinese (nine females and eight males, age M = 26, SD = 2.1) participated in Experiment 1. All of them were without prior knowledge of the speech stimuli (detailed in the following section). They reported no hearing or speech impairments.

#### Procedure

Participants were tested individually in a sound-attenuated booth. The experiment was presented and controlled using Python 3.5 on a Lenovo computer running Windows 10. The stimuli were presented in two blocks, 10 tokens of each vowel per block. The female version of the speech targets was presented to female participants while the male version of the speech targets was presented to male participants. Participants were asked to reproduce the sound after hearing each auditory token of the speech targets (10 tokens per speech target, via Siemens HD280 headphones). They had 4000 ms to complete the production and recording of each sound token. The auditory feedback of participants’ own voice was manipulated in two conditions: masked with noise (the *masked* condition) and without noise (the *unmasked* condition). They completed the *masked* condition first before doing the *unmasked* condition. Pink noise generated in MATLAB (2014a) was used as the masking noise due to its effectiveness for masking speech^53^. The noise lasted 4000 ms with constant sound pressure level throughout. The intensity of the noise was adjusted according to each participant’s response so that it was presented at a relatively comfortable level with sufficient loudness to mask their own voice feedback.

#### Data analyses

The following acoustic parameters were extracted from Praat^54^: F1 (the first formant of the vowel) and F2 (the second formant of the vowel). Formant trajectory tracking in Praat was manually corrected where necessary. Following Flege *et al*.^55^, Hertz was converted to Barks (B1 and B2 respectively) to minimize male vs. female differences. The extent to which non-native participants’ production of /ø/ and /ɵ/ differed from native speakers’ production of the vowels was measured by the Euclidean distance of each participant’s B1 and B2 values from the native speakers’ B1 and B2 values. Therefore, the dependent variable of the present study was the Euclidean distance between the learned and target vowels.

#### Results

**Figure 2:**
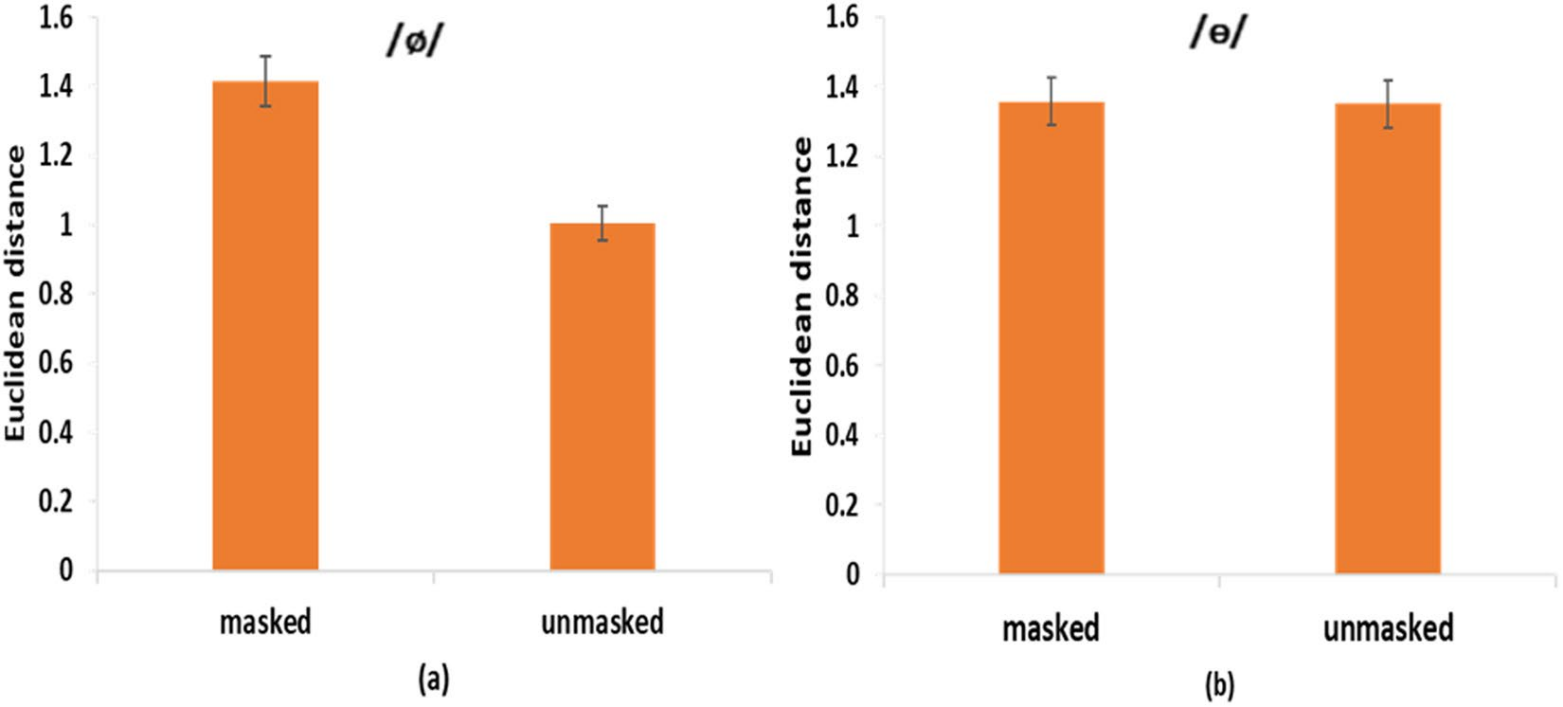
Speech production results of Experiment 1: (**a**) /ø/ Euclidean distance and (**b**) /ɵ/ Euclidean distance in *masked* and *unmasked* conditions. Error bars represent standard error of the mean.

**Figure 3:**
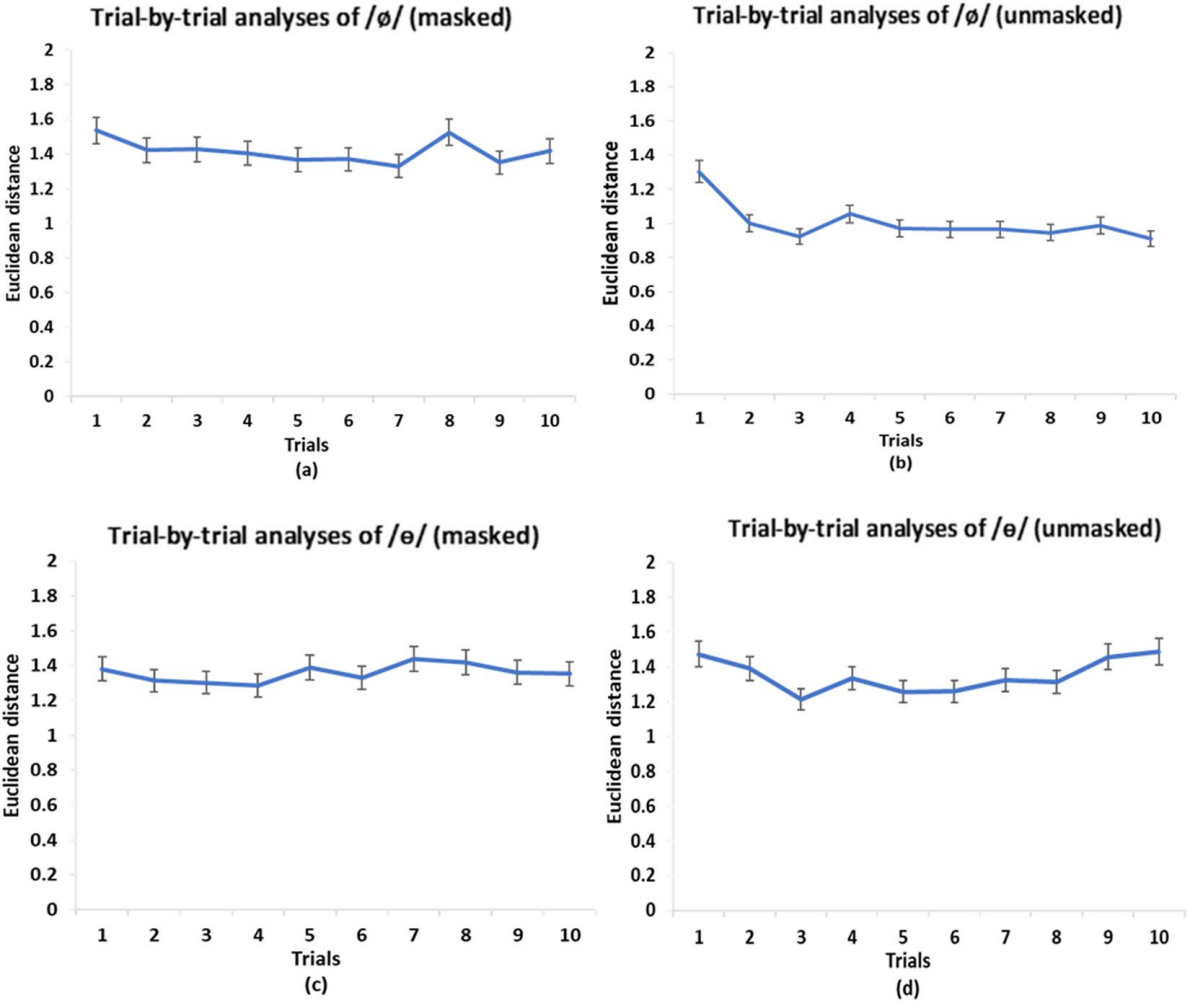
Mean values of the Euclidean distance of each trial (10 trials in total) of the production of /ø/ and /ɵ/ by all the participants in the *masked* (**a**) (**c**) and *unmasked* (**b**) (**d**) conditions. Error bars represent standard error of the mean.

Figure 2(a,b) show the Euclidean distance between the learned and target vowels (/ø/ and /ɵ/) in the two auditory feedback conditions. A two-way (vowels and auditory feedback conditions) repeated measures ANOVA showed a significant main effect of auditory feedback conditions [*F* (1, 16) = 9.35, *p* = 0.008, *η*^2^_p_ = 0.37], and interaction between the two factors [*F* (1, 16) = 4.58, *p* = 0.048, *η*^2^_p_ = 0.22]. For /ø/, a following one-way (*masked* vs. *unmasked*) repeated measures ANOVA showed a significant main effect of availability of auditory feedback [*F* (1, 16) = 8.53, *p* = 0.01, *η*^2^_p_ = 0.35]. Specifically, the Euclidean distance in the *unmasked* condition was smaller (*M* = 1.00, *SEM* = 0.16) than that in the *masked* condition (*M* = 1.42, *SEM* = 0.18). Furthermore, follow-up trial-by-trial analyses showed that for the feedback *masked* condition (Fig. 3a), there was not a significant difference between the highest-value trial and the lowest-value trial [*F* (1, 16) = 1.23, *p* = 0.28]. For the feedback *unmasked* condition (Fig. 3b), however, the difference between the highest-value trial and lowest-value trial was significant [*F* (1, 16) = 8.05, *p* = 0.01, *η*^2^_p_ = 0.34]. These results suggest that vocal learning performance on /ø/ was better in the *unmasked* condition than in the *masked* condition.

For /ɵ/, the *masked* condition showed a greater distance from the standard speech target (*M* = 1.36, *SEM* = 0.11) than the *unmasked* condition (*M* = 1.35, *SEM* = 0.09). However, a one-way repeated measures ANOVA showed that there was not a significant main effect of auditory feedback on the Euclidean distance [*F* (1, 16) = 0.007, *p* = 0.94]. Furthermore, a one-way repeated measures ANOVA showed that for both feedback *masked* and *unmasked* conditions (Fig. 3c,d), there was not a significant difference between the highest-value trial and lowest-value trial [*F* (1, 16) = 2, *p* = 0.18 for the *masked* condition; *F* (1, 16) = 2.21, *p* = 0.16 for the *unmasked* condition]. These findings suggest that vocal learning performance on /ɵ/ was equally bad in both the *masked* and *unmasked* conditions.

## Experiment 2

### Methods

The study was approved by NYU Shanghai Research Ethical Committee. All experiments were performed according to relevant guidelines and regulations. Informed consent was obtained from all participants for the experiments. Data sets are accessible from https://osf.io/cnvh7/.

#### Participants

Forty adult speakers of Mandarin Chinese (twenty females and twenty males, age *M* = 26, *SD* = 1.7) different from Experiment 1 participated in Experiment 2. They did not have prior knowledge of the two vowels. They reported no hearing or speech impairments.

#### Stimuli

The stimuli were the same as Experiment 1.

#### Procedure

**Figure 4:**
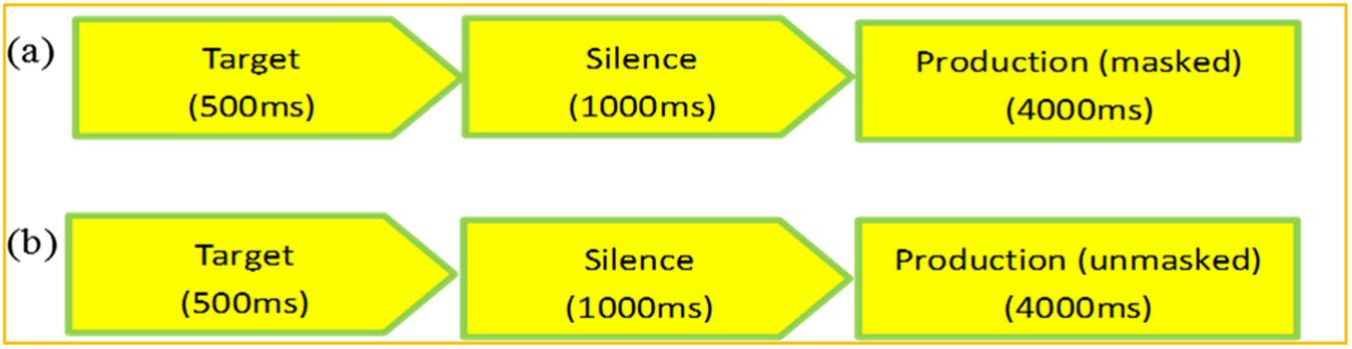
Schematic illustration of the two conditions (**a**: masked; **b**: unmasked) of the speech production task.

As mentioned in the introduction, Experiment 2 uses a between-subject design with two additional listening control tasks, with the aims to: 1) test whether Experiment 1 can be replicated; 2) control for practice effect which could result from a within-subject design; 3) control for perception and memory accuracy which could affect participants’ speech production performance in this study, with two listening control tasks (detailed below). Twenty participants (ten females and males) were tested with their auditory feedback masked with noise (the *masked* condition) and the other twenty participants (ten females and males) were tested with the feedback unmasked (the *unmasked* condition). Consistent with Experiment 1, participants were tested individually in a sound-attenuated booth. The experiment was presented and controlled using Python 3.5 on a Lenovo computer running Windows 10. The stimuli were presented in two blocks for the two vowels respectively, with 10 tokens per block. The audio presentation (via Siemens HD280 headphones) of each token of the sound was followed by a 1000 ms silence interval, after which participants were prompted by a cross (“+”) on the computer screen to repeat the speech target they just heard. They had 4000 ms to complete the recording of each token of the sound. For the *masked* condition, the noise started at the same time as the appearance of the cross on the screen and lasted 4000 ms. The vocal reaction time (i.e., the time interval between the initial appearance of the cross on the screen and the onset of the participant’s voice production) was also recorded. A schematic illustration of the speech production task is presented in Fig. 4.

**Figure 5:**
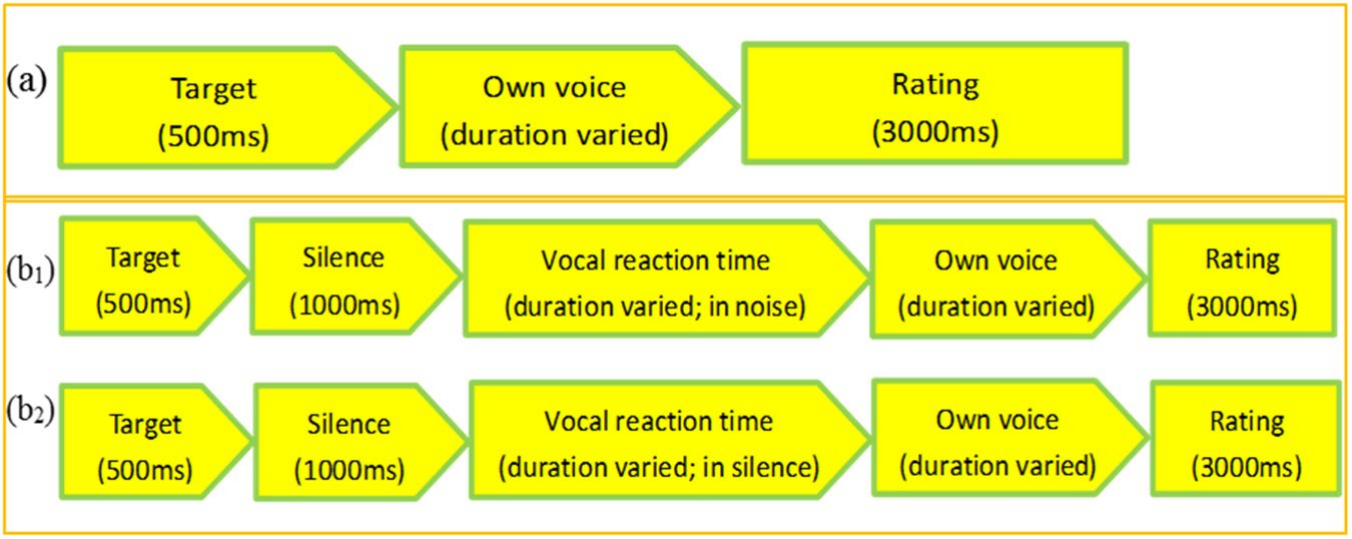
Schematic illustration of the two listening control tasks. (**a**) The listening task 1 (perception task). The listening task 2 (memory task): the upper (**b1**) and lower row (**b2**) represent the masked and unmasked condition respectively.

Two listening tasks were carried out after the speech production task. The listening task 1 (perception task) aimed to test whether participants had good perception of the target speech stimuli. All forty participants listened to the target speech sounds immediately followed (i.e., without silence interval) by their own production of the sounds which varied in duration among participants. They were asked to judge on a scale of 1–3 on a keyboard how well they produced the target sounds: 1 = good; 2 = average; 3 = bad. They had 3000 ms to complete the ratings. It was followed by listening task 2 (memory task) which aimed to test whether participants had good memory of the target speech stimuli. The procedure was the same as listening task 1 except that there was a time interval between the presentation of the target sound and the learned sound, which lasted as long as 1000 ms (silence) plus the vocal reaction time (varied across trials and between individuals) recorded from the speech production task. In addition, for the twenty participants under the *masked* condition, the same masking noise as in the speech production task was presented during the vocal reaction time in listening task 2. The rationale behind listening task 2 is to fully restore the background listening condition of the speech production task so that the influence of memory could be tested. For each listening task, there were 10 trials of /ø/ and 10 trials of /ɵ/ (recorded from each participant’s speech production task). Figure 5 is a schematic illustration of the procedure of the two listening tasks.

#### Data analyses

Data analyses for production of the two vowels were the same as Experiment 1. In addition, participants’ ratings of how well their speech production compared with the target sounds in the two listening tasks were also analyzed using Pearson correlation tests.

#### Results

**Figure 6:**
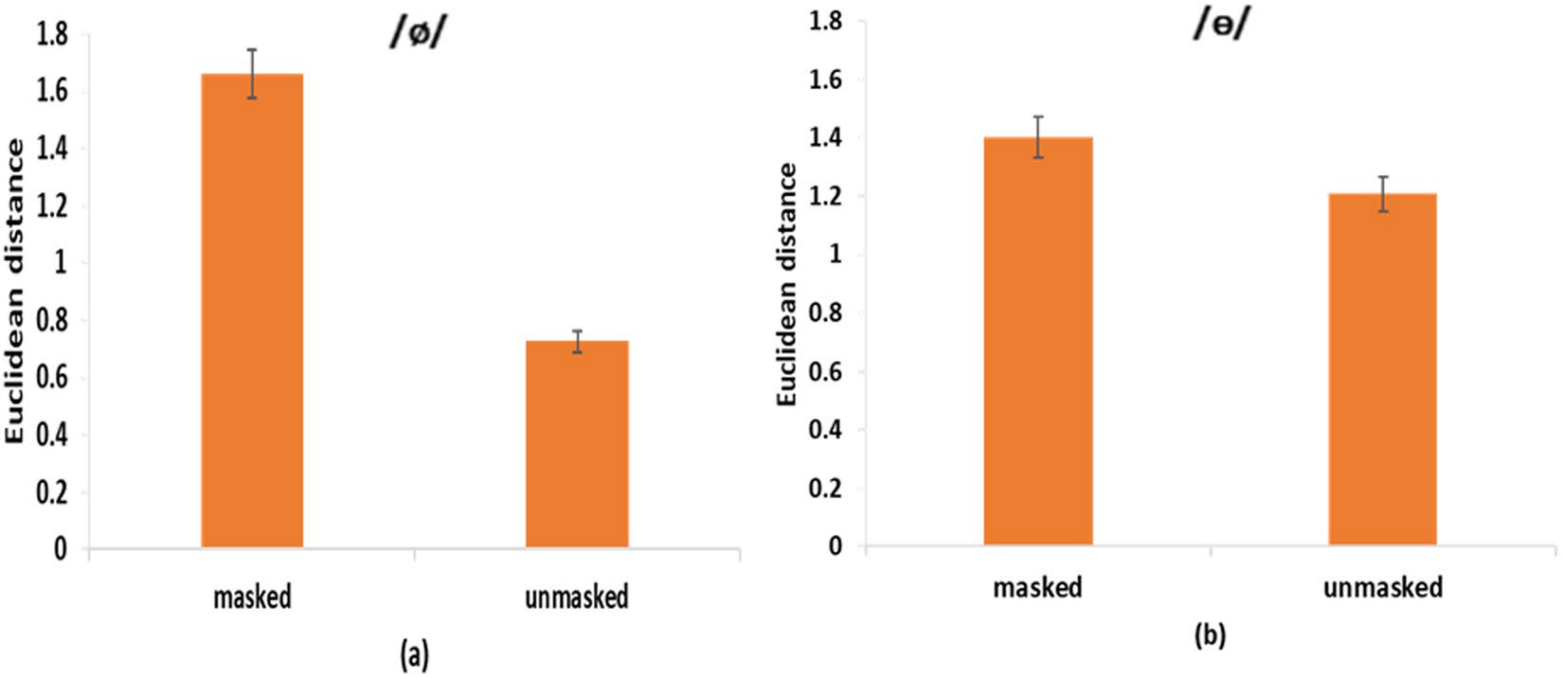
Speech production results of Experiment 2: (**a**) /ø/ Euclidean distance and (**b**) /ɵ/ Euclidean distance in *masked* and *unmasked* conditions. Error bars represent standard error of the mean.

**Figure 7:**
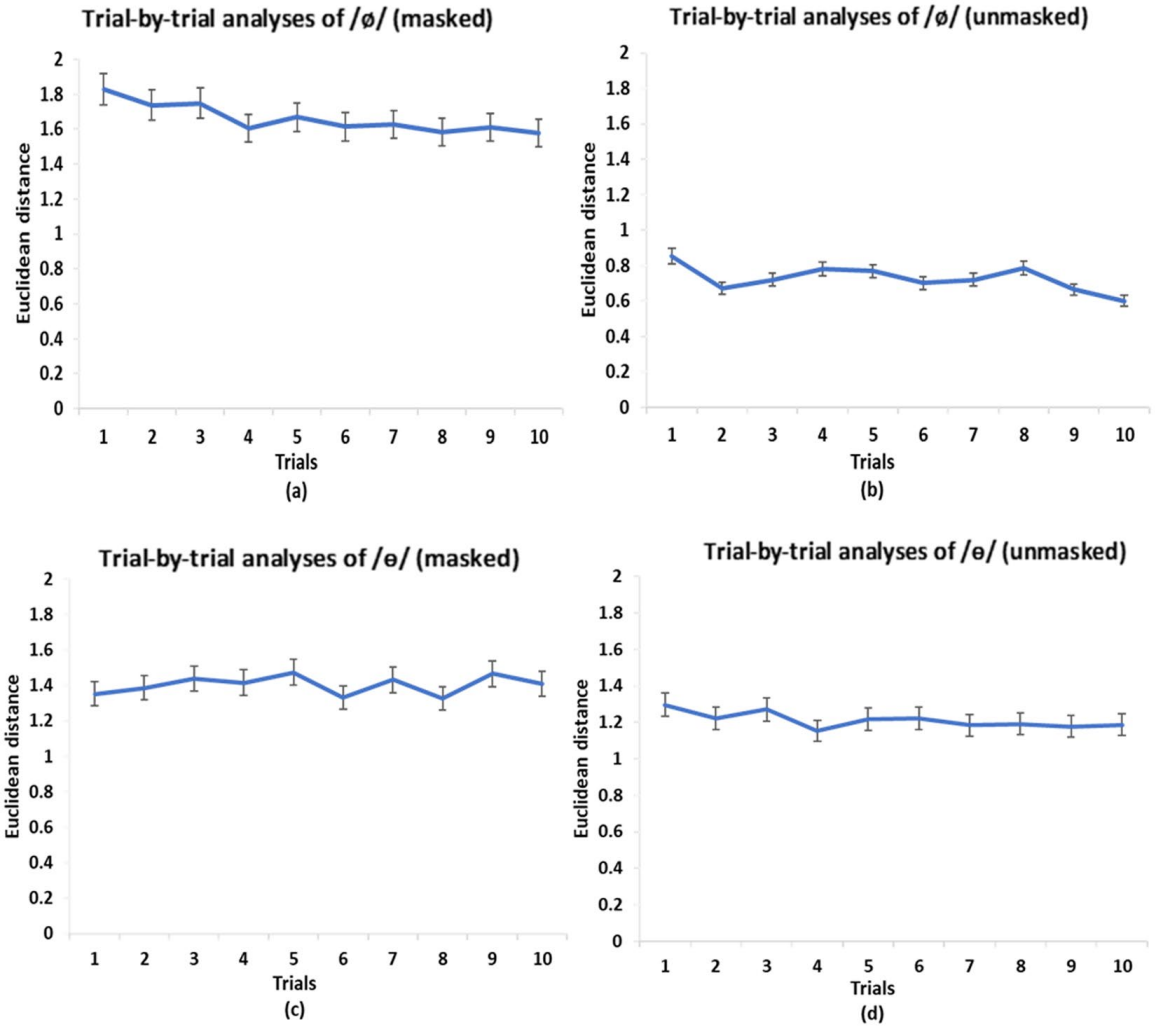
Mean values of the Euclidean distance of each trial (10 trials in total) of the production of /ø/ and /ɵ/ by all the participants in the *masked* (**a**) (**c**) and *unmasked* (**b**) (**d**) conditions. Error bars represent standard error of the mean.

Figure 6(a,b) show the Euclidean distance between the learned and target vowels (/ø/ and /ɵ/) in the two auditory feedback conditions. A two-way (vowels and auditory feedback conditions) mixed ANOVA showed a significant main effect of auditory feedback conditions [F(1, 38) = 19.8, *p* < 0.001, *η*^2^_p_ = 0.34], and interaction between vowels and auditory feedback conditions [F(1, 38) = 23.89, *p* < 0.001, *η*^2^_p_ = 0.39]. For /ø/, a follow-up one-way (*masked* vs. *unmasked*) between-subjects ANOVA showed a significant main effect of availability of auditory feedback [*F* (1, 38) = 54.29, *p* < 0.001]. Specifically, the Euclidean distance in the *unmasked* condition (*M* = 0.73, *SEM* = 0.07) was smaller than that in the *masked* condition (*M* = 1.66, *SEM* = 0.11). Furthermore, a one-way repeated measures ANOVA showed that for the feedback *masked* condition (Fig. 7a), there was not a significant difference between the highest-value trial and lowest-value trial [*F* (1, 19) = 3.67, *p* = 0.07]. For the feedback *unmasked* condition (Fig. 7b), however, the difference between the highest-value trial and lowest-value trial was significant [*F* (1, 19) = 7.71, *p* = 0.01, *η*^2^_p_ = 0.29]. These results suggest that the vocal learning performance on /ø/ was better in the *unmasked* condition than in the *masked* condition.

For /ɵ/, the *masked* condition showed higher distance from the standard speech target (*M* = 1.35, *SEM* = 0.15) than the *unmasked* condition (*M* = 1.21, *SEM* = 0.1). However, similar to experiment 1, a one-way (*masked* vs. *unmasked*) between-subjects ANOVA showed that there was not a significant main effect of auditory feedback [*F* (1, 38) = 0.68, *p* = 0.41]. Furthermore, a one-way repeated measures ANOVA showed that for both feedback *masked* and *unmasked* conditions (Fig. 7c,d), there was not a significant difference between the highest-value trial and lowest-value trial [*F* (1, 19) = 0.8, *p* = 0.38 for the *masked* condition; *F* (1, 19) = 2.19, *p* = 0.16 for the *unmasked* condition]. These findings suggest that the vocal learning performance on /ɵ/ was equally bad in both the *masked* and *unmasked* conditions.

**Figure 8:**
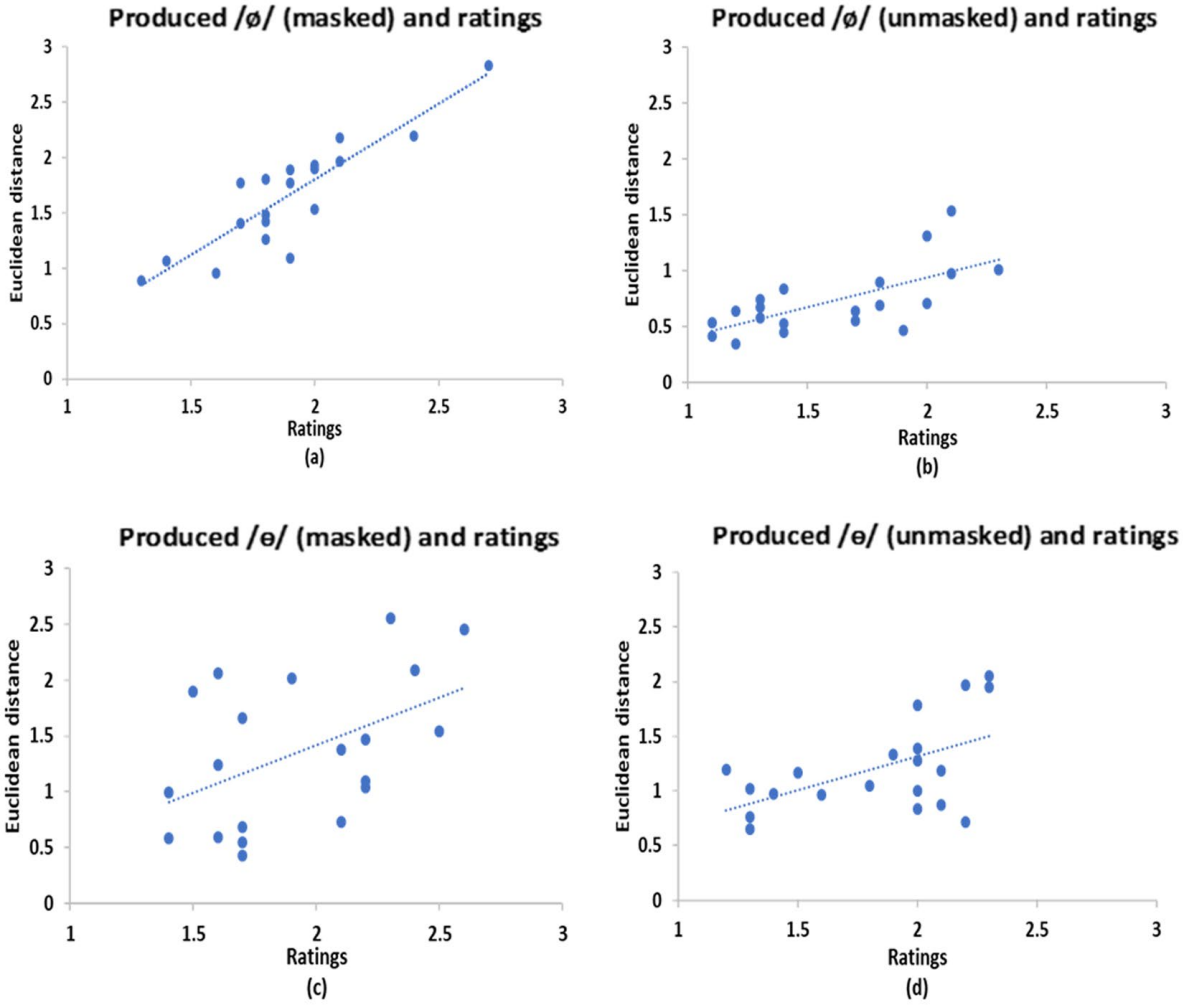
Results of listening task 1 (perception task) in Experiment 2: scatter plots of correlations between ratings (1 = good; 2 = average; 3 = bad) and the Euclidean distance between learned and target production of /ø/ and /ɵ/ (**a**,**c**: *masked*; **b**,**d**: *unmasked*).

To rule out the potential confound of auditory perceptual ability, a Pearson correlation was run to determine the relationship between participants’ produced speech and their corresponding ratings in the listening task 1 (perception task). The scatter plots (Fig. 8) summarize the results. For /ø/, there was significantly positive correlations between the Euclidean distance and ratings for the two auditory feedback conditions (*r* = 0.88, *n* = 20, *p* < 0.001 for the *masked* condition; *r* = 0.67, *n* = 20, *p* = 0.001 for the *unmasked* condition). Similar results were obtained for /ɵ/, i.e., significant positive correlations were found between the Euclidean distance and ratings (*r* = 0.48, *n* = 20, *p* = 0.03 for the *masked* condition; *r* = 0.54, *n* = 20, *p* = 0.01 for the *unmasked* condition). The results suggest participants had good perception of the target speech stimuli as they could differentiate between good and bad sound tokens produced by themselves based on comparison with the target speech stimuli.

**Figure 9:**
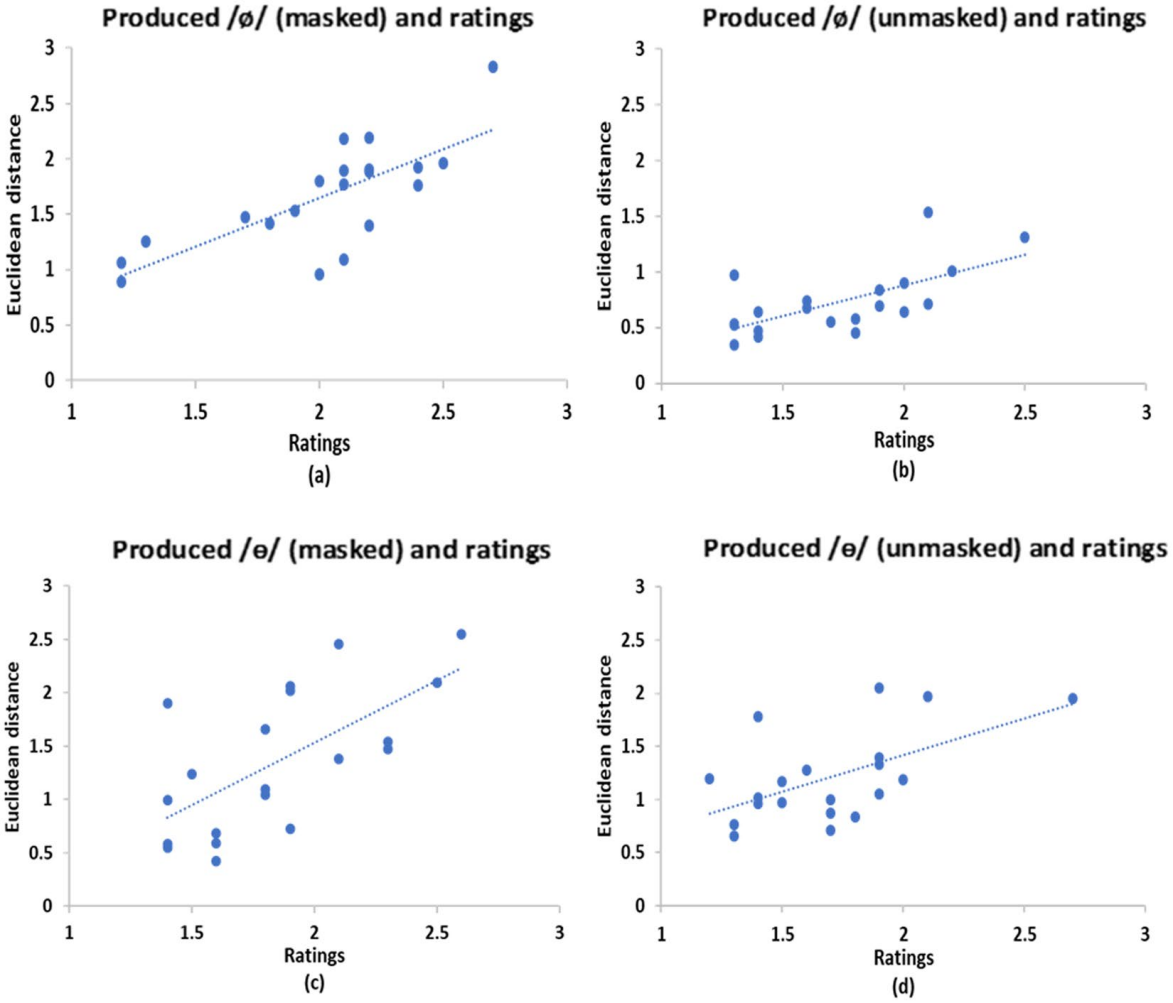
Results of listening task 2 (memory task) in Experiment 2: scatter plots of correlations between ratings (1 = good; 2 = average; 3 = bad) and the Euclidean distance between learned and target production of /ø/ and /ɵ/ (**a**,**c**: *masked*; **b**,**d**: *unmasked*).

To rule of the potential confound of auditory memory, another Pearson correlation was run to determine the relationship between participants’ produced speech and their corresponding ratings in the listening task 2 (memory task). The scatter plots (Fig. 9) summarize the results. For /ø/, there were significant and positive correlations between the Euclidean distance and ratings for both auditory feedback conditions (*r* = 0.75, *n* = 20, *p* < 0.001 for the *masked* condition; *r* = 0.66, *n* = 20, *p* = 0.002 for the *unmasked* condition). Similar results were obtained for /ɵ/, i.e., significant positive correlations were found between the Euclidean distance and ratings (*r* = 0.66, *n* = 20, *p* = 0.002 for the *masked* condition; *r* = 0.57, *n* = 20, *p* = 0.009 for the *unmasked* condition). The results suggest participants had good memory of the target speech stimuli, because the lapse of time (interval) and the acoustic noise introduced in the main experiment did not prevent them from being able to differentiate between good and bad sound tokens produced by themselves based on comparison with the target speech stimuli.

## Discussion

In the present study, we investigated an important but less-studied aspect of speech motor control: whether prediction can compare directly with goals in adults’ acquisition of L2 speech sounds with different degrees of similarity to L1. Adult native Mandarin speakers learned two types of vowels: /ø/ and /ɵ/. The former is less similar than the latter to Mandarin Chinese vowels. Online auditory feedback during participants’ production of the two vowels was manipulated in two conditions: feedback masked with noise (the *masked* condition) and feedback without noise (the *unmasked* condition). The results were consistent between the two experiments (within- and between-subjects designs respectively): acoustic differences between the learned sounds and target sounds did not show significant improvement in the *masked* condition. In addition, the presence of auditory feedback in the *unmasked* condition was effective in improving learning of /ø/, not /ɵ/. These results support our hypotheses that prediction and goals cannot be directly compared in adult L2 speech learning, regardless of the extent of similarity between L1 and L2 sounds. On the other hand, when auditory feedback becomes available for speech learning, the L1 vs. L2 speech target similarity plays a significant role in deciding learning performance.

The two listening control tasks for perception and memory respectively showed that the larger the deviation between the learned and target speech (indexed by Euclidean distance between the target and learned vowels), the higher the ratings (1 = good, 2 = average, 3 = bad). That is, bad ratings reflect larger deviation from the target speech. This suggests that participants had good perception (revealed in listening task 1) and memory (revealed in listening task 2), since they could differentiate between good and bad speech tokens produced by themselves in the two listening tasks where perception and memory were tested respectively. Therefore, we can rule out the possibility that bad perception and memory could affect the speech production performance observed in this study.

The results on feedback masked condition suggest that without auditory feedback, prediction is not sufficient to achieve sensory goals in adult L2 acquisition, irrespective of how similar L2 to L1 sounds are. The reason could be that unlike infants whose motor-sensory transformation in speech production is yet to be fully formed, adults have already had well-established representation for motor-sensory transformation due to long-term native language practice^40,41^. Therefore, when learning L2 sounds, adults tend to utilize the causal relation between motor commands and sensory output in their native language to approximate the L2 speech targets^56^. That is why in L2 perception and production, a carry-over effect of phonetic norms of L1 to L2 (i.e., phonetic interference) always exists^57^. As a result, accurate pronunciation of the L2 targets requires adult learners to overcome well-established articulatory habits for L1 in order to adjust to the motor commands of L2 targets^58^. The present study further shows that such adjustment of the motor commands for L2 needs the regulation of auditory feedback, as the results showed that when feedback was masked, motor prediction alone cannot successfully implement the L2 targets, i.e., prediction cannot compare directly with goals.

The results on feedback unmasked condition showed that feedback was effective in improving learning of /ø/, not /ɵ/. This suggests that although feedback is important in speech acquisition^8,29^, the effectiveness of feedback in speech acquisition could be conditional upon the type of target speech sounds, especially in adult L2 acquisition. As shown in this study, feedback is not always effective when the novel speech sound is in perception and production similar to an existing sound in adults’ native speech inventory. The reasons could be twofold. Firstly, it is known that auditory feedback is subject to delays caused by synaptic transmission and central processing^2^. Therefore, relying on sensory feedback for correction of online motor movements may lead to instability or even errors^59^. That is why motor prediction realized through an internal forward model^1,7^ is also needed to counteract sensory delays in predicting the current state of motor movement^14^. This also explains why motor-based prediction is the main mechanism underlying mature speech production^29^, because the delay in auditory feedback is likely to restrict the effective control of fluent speech production, especially given that average speech rate can be as fast as 10 syllables/second. Secondly, as pointed out in Flege^37^, the more similarities between the L1 and L2 sounds, the more difficult it is to master the L2 sound. In this study, /ɵ/ is close to (although still distinct from) Mandarin vowel /ɤ/. Such similarity could prevent learners from effectively establishing an accurate motor representation of /ɵ/. That is, it is the prior establishment of the motor representation of Mandarin /ɤ/ that prevents the successful motor-based prediction and implementation of /ɵ/ due to articulatory and perceptual closeness between the two sounds. Therefore, it could be inferred that the online production of /ɵ/ by participants in this study was probably guided by the prediction for Mandarin /ɤ/ which is a mature, well-represented speech sound for Mandarin speakers. This, plus the relatively ineffective auditory feedback control of mature speech production discussed above, leads to the result that learning /ɵ/ was equally unsuccessful regardless of whether auditory feedback was masked or not.

The present study contributes to a better understanding of sensorimotor integration in speech production through the relations between feedback, prediction and goals in adult L2 acquisition. As introduced in section 1.3, previous studies on sensorimotor integration in speech production^2,5,26,50^ are more focused on the relations between feedback vs. goals (e.g., children’s acquisition of native speech) and feedback vs. prediction (e.g., self-induced speech suppression; feedback perturbation). The present study could shed light on the less-studied aspect: prediction vs. goals. When feedback was not available, no improvement in learning of the two speech sounds was observed. From a sensorimotor perspective, this further suggests that motor prediction and sensory goals cannot be directly compared in adult L2 acquisition. The reason could be that for adults trying to learn novel L2 speech sounds without the availability of feedback, the neural demand for forming the precise prediction for novel speech output would compete against the demand for maintaining goals for learning. It is thus possible that the formation of prediction could overtake the neural resources originally for maintaining goals, thus rendering prediction incomparable with goals. When L1 and L2 target sounds are too similar, prediction could more easily overwrite the goal due to the mature prediction established from L1 motor-sensory loop, thus rendering auditory feedback less effective in updating the sensorimotor transformation for the novel L2 speech targets (goals). The reason for our proposal of such competing neural relations between prediction and goals in adult L2 learning could be found in the neural mechanism of speech production. As has been suggested in previous studies^58,60,61^, the neural representation of speech production could boil down to a multisensory motor representation that contains various information such as language-specific sensory targets, individual vocal tract configuration and auditory feedback. As a result, it is reasonable to infer that motor prediction and sensory goals may share a similar neural representational format, and hence could compete for neural resources during the process of learning to articulate a non-native speech sound.

## Conclusion

Using an auditory feedback masking paradigm, the present study shows that prediction via motor-sensory transformation is not directly comparable with sensory goals in adult L2 acquisition. Furthermore, auditory feedback can help achieve learning only if the competition between prediction and goals is minimal, i.e., when target sounds are distinct from existing sounds in one’s native speech inventory. Our results suggest that prediction and goals may share a similar representational format, which could result in a competing relation in neural recourses that constrains speech learning. The feedback can conditionally overcome such interference between prediction and goals. Hence, through the lens of adult L2 acquisition, the present study sheds new light on the functional relations among key components (prediction, goals and feedback) of sensorimotor integration in speech learning.

### Data Availability

The datasets for the current study are accessible from https://osf.io/cnvh7/.
